# Quinoa dough fermentation by *Saccharomyces cerevisiae* and lactic acid bacteria: Changes in saponin, phytic acid content, and antioxidant capacity

**DOI:** 10.1002/fsn3.3679

**Published:** 2023-09-14

**Authors:** Sanaz Arjmand, Neda Mollakhalili‐Meybodi, Fateme Akrami Mohajeri, Farzan Madadizadeh, Elham Khalili Sadrabad

**Affiliations:** ^1^ Research Center for Food Hygiene and Safety Department of Food Hygiene and Safety, School of Public Health Shahid Sadoughi University of Medical Sciences Yazd Iran; ^2^ Research Center for Food Hygiene and Safety Department of Food Science and Technology, School of Public Health Shahid Sadoughi University of Medical Sciences Yazd Iran; ^3^ Infectious Diseases Research Center Shahid Sadoughi Hospital Shahid Sadoughi University of Medical Sciences Yazd Iran; ^4^ Center for Healthcare Data modeling Departments of Biostatistics and Epidemiology, School of public health Shahid Sadoughi University of Medical Sciences Yazd Iran

**Keywords:** antioxidant capacity, lactic acid bacteria, quinoa flour, saponin and phytic acid

## Abstract

The effects of two fermentation processes (common fermentation with *Saccharomyces cerevisiae* and fermentation by *Lacticaseibacillus casei* subsp. *casei* PTCC 1608 and *Lactiplantibacillus plantarum* subsp. *plantarum* PTCC 1745) on pH, titratable acidity, total phenolic and flavonoid contents, antioxidant capacity, saponin content, as well as phytic acid content of quinoa dough were investigated during the 24‐h fermentation (4‐h interval). According to the results, the highest titratable acidity was observed in the samples fermented by *L. casei* subsp. *casei*. Moreover, the highest antioxidant capacity was observed after 12 h of fermentation by *L. plantarum* subsp. *plantarum* (31.22% for DPPH, 104.67% for FRAP) due to a higher concentration of phenolic compounds produced (170.5% for total phenolic content). Also, all samples have been able to reduce saponin by 67% on average. Furthermore, the samples fermented by *L. plantarum* subsp. *plantarum* showed the most significant decrease in phytic acid content (64.64%) during 24‐h fermentation. By considering the reduction of the antinutritional compounds and improvement in the antioxidant properties of quinoa flour, the *Lactiplantibacillus plantarum* strain was recommended.

## Graphical abstract

The effects of two fermentation processes (common fermentation with Saccharomyces cerevisiae and fermentation using *L. casei* subsp. casei PTCC 1608 and *L. plantarum* subsp. *plantarum* PTCC 1745) on pH, titratable acidity, total phenolic and flavonoids contents, antioxidant capacity, saponin content, as well as phytic acid content of quinoa dough were investigated during the 24‐h fermentation in 4‐h interval.

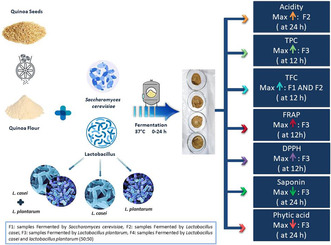

## 1 INTRODUCTION

Quinoa (*Chenopodium quinoa* Willd) is a gluten‐free pseudo‐cereal native to the Andes region of South America (Nickel et al., 2016). It has been cultivated in different geographical conditions due to its high resistance to various environmental conditions (drought, frost, salinity, water shortage, and acid soil) (Hernández‐Ledesma, 2019). Nowadays, increased attention has been devoted to quinoa as a highly nutritional grain, containing a high quantity of protein (with balanced essential amino acids), vitamins, lipids (unsaturated fatty acids), minerals, and other beneficial compounds (Vilcacundo & Hernández‐Ledesma, 2017). Regarding this, it has been introduced as vegetarian caviar by the Food and Agriculture Organization (FAO) (Zandi et al., 2020). Quinoa is well known due to the presence of many lipophilic and hydrophilic bioactive compounds (Fernández‐López et al., 2020) including tocopherols, carotenoids as well as polyphenols, and betacyanins which are potentially related to its antioxidant activities (Bastidas et al., 2016; Fernández‐López et al., 2020). Antioxidants can prevent cancer, allergies, Alzheimer's, and cardiovascular diseases (Tang et al., 2015). Despite its nutritious nature, quinoa is also considered a rich source of antinutritional compounds including saponin and phytic acid (Vega‐Gálvez et al., 2010).

Saponin is known as the main antinutritional compound in quinoa which limits the bioavailability of vitamins and minerals by forming complexes and preventing their absorption in the small intestine (Gemede & Ratta, 2014). The widespread usage of quinoa is also limited owing to its bitter taste, which is attributed to the presence of saponin. The saponin of quinoa is classified in the group of triterpenoid saponins. Triterpenoids consist of a linear arrangement of 1–6 hexose or pentose glucoside units (the sugar moiety) linked to the sapogenin aglycone (the nonsugar moiety) through glycosidic linkages (such as β‐glucoside) (Ruales & Nair, 1993). So far, washing, pearling, germination, and fermentation are considered the main methods that have been used to reduce saponins (Suárez‐Estrella et al., 2018). As saponin has a glycosidic bond (such as β‐glucoside) in its structure, glucosidase enzymes such as β‐glucosidase are also considered effective (Michlmayr & Kneifel, 2014).

Phytic acid, which is generally known as myo‐inositol‐1,2,3,4,5,6‐hexakis‐dihydrogen phosphate, is distributed in the endosperm of quinoa and its outer layers (Jancurová et al., 2009). Phytic acid can form insoluble complexes with essential dietary minerals such as iron, zinc, calcium, and magnesium to prevent their bioavailability in the body (Samtiya et al., 2020). It can also inhibit the activity of vital enzymes in the digestive system such as α‐amylase, trypsin, lipase, phosphatase, and pepsin which leads to insufficient digestion and absorption of nutrients (KS et al., 2021). Therefore, different strategies are used to reduce their content, including soaking, sprouting, enzymatic treatment with phytase, and fermentation (Gupta et al., 2015). Regarding the efficiency of the fermentation process, which has also widely been recognized as a source of β‐glucosidase enzyme inoculation, the reduction of saponin and phytic acid could be investigated (Singhania et al., 2013).

Fermentation is a food production and preservation method in which microorganisms are used to convert carbohydrates into an alcohol or acid (Maicas, 2020). The benefits of the fermentation process include increasing the levels and bioavailability of nutrients, reducing antinutrient compounds, and improving of taste, texture, and smell of the final product (Blandino et al., 2003). Also, it has been claimed to improve the antioxidant capacity of the fermented matrix (Hur et al., 2014). The main microorganisms used in the fermentation process include common yeast (*Saccharomyces cerevisiae*) and lactic acid bacteria (Blandino et al., 2003; Maicas, 2020). Considering the importance of β‐glucosidase activity in its potential efficiency in saponin reduction (Son et al., 2018), *Lacticaseibacillus casei* and *Lactiplantibacillus plantarum* are considered potentially efficient strains with β‐glucosidase activity (Ávila et al., 2009). Due to the nutritional importance of quinoa and concerns about its saponin and phytic acid content, the current study aimed to investigate the efficiency of two fermentation processes (common fermentation with *Saccharomyces cerevisiae* and fermentation by *Lacticaseibacillus casei* and *Lactiplantibacillus plantarum*) in the removal of saponin and phytic acid and also improvement of antioxidant capacity.

## 2 MATERIALS AND METHODS

### 2.1 Materials and chemical reagent

The gallic acid, phytic acid sodium, 2,2‐Diphenyl‐1‐picrylhydrazyl (DPPH), 2,4,6‐tris (2‐pyridyl)‐s‐triazine (TPTZ) were purchased from Sigma‐Aldrich, while saponin, Folin–Ciocalteu reagent, phenolphthalein reagent, sodium hydroxide, sodium acetate trihydrate, hydrochloric acid, iron (III) chloride hexahydrate, sulfosalicylic acid, glacial acetic acid, sulfuric acid, methanol, ethanol, sodium carbonate were obtained from Merck. The de‐saponifying quinoa grains (removed by soaking and mechanical abrasion) were purchased from Ilia Trade Development Company. The grains were milled using a grinder, and the samples collected between mesh sizes of 50 and 100 were used for further analysis. *Saccharomyces cerevisiae* yeast was obtained from Iran mayeh Company™, *Lacticaseibacillus casei* subsp. *casei* PTCC 1608 and *Lactiplantibacillus Plantarum* subsp. *Plantarum* PTCC 1745 was obtained from Zist Takhmir Company™.

### 2.2 Microorganisms and culture conditions

The lactic acid bacteria (LABs) including *Lacticaseibacillus casei* subsp. *casei* PTCC 1608 and *Lactiplantibacillus plantarum* subsp. *plantarum* PTCC 1745 (0.5 mL) were cultured in MRS broth media (50 mL) according to the method described by Dallagnol et al with some modifications. The cultured bacteria were incubated at 37°C for 24 h (Chiș et al., 2018).

### 2.3 Fermentation process

Approximately 120 g of quinoa flour was poured into 150 mL of sterile distilled water, and then 2.2% (W/W) of *Saccharomyces cerevisiae*, and Lactic strains (*L. casei* and *L. plantarum*), or the combination of *L. casei* and *L. plantarum* were used. The samples were divided into four groups including F1: the samples fermented by *Saccharomyces cerevisiae*; F2: the samples fermented by *L. casei*; F3: the samples fermented by *L. plantarum*; and F4: the samples fermented by the combination of *L. casei* and *L. plantarum* strains. The fermentation was done in an incubator at 37°C for 24 h. The samples were taken over 4‐h intervals under aseptic conditions for further analysis. The samples were dried in an oven at 60°C for 12 h and were stored in a −20°C refrigerator (Hashemi et al., 2019).

### 2.4 Analytical methods

#### 2.4.1 Determination of titratable acidity

The total titratable acidity was determined according to the Rizzello et al method. Accordingly, 10 g of samples was dissolved in 90 mL of distilled water and titrated by NaOH (0.1 M) in the presence of phenolphthalein. The total titratable acidity was calculated by the NaOH volume used (Rizzello et al., 2016).

#### 2.4.2 Extraction of samples

The extraction of samples for determination of the total phenolic, flavonoid, and antioxidant capacity was done according to the method explained by Alvarez‐Jubete et al. (2010) with some modifications. The fermented sample (0.5 g) was added to 5‐mL methanol and the solution was stirred for 2 h and then centrifuged at 2000 × *g* for 10 min. The supernatant solution was separated and diluted with methanol to reach a volume of 25 mL. The extracted samples were stored in the refrigerator (−20°C) until further analysis (Alvarez‐Jubete et al., 2010).

#### 2.4.3 Total phenolic content

Total phenolic content was measured using the Folin–Ciocalteu method. About 500 μL of the extracted sample was mixed with 10 times diluted Folin–Ciocalteu reagent (2.5 mL), after 6 min, 2 mL of Na_2_Co_3_ 7.5% was added and left at room temperature for 60 min. The absorbance of the samples was measured using a spectrophotometer at 765 nm. The total amount of phenolic compounds was expressed as milligrams of gallic acid equivalent per 100 g of dry sample (mg GAE/100 g) (Sarafraz Ardakani et al., 2023).

#### 2.4.4 Determination of total flavonoid content

In order to determine the flavonoid content, 250 μL of the extracted samples was added to 1.25 mL of deionized distilled water and 75 μL of sodium nitrite (5% V/W) and remained for 6 min. Then, 150 μL of aluminum chloride hydrate was added to the solution. After 5 min, 0.5 mL of sodium hydroxide solution (1 M) and 275 μL of ethanol were added to the solution and the absorbance of the samples was measured at 510 nm against distilled water as a blank. The total amount of flavonoid content was expressed as milligrams of rutin equivalent per 100 g of dry sample (mg rutin/100 g) (Amiri et al., 2023).

#### 2.4.5 DPPH assay

The DPPH assay was done by the addition of 2.5 mL of 2, 2‐diphenyl‐1‐picrylhydrazyl (DPPH) methanolic solutions to 0.5 mL sample extracted. The absorbance of samples was recorded after 30 min at 517 nm against methanol. The radical scavenging activity of DPPH was calculated as follows:

$$\left(\%\right)\;\text{radical scavenging activity}:\left[\left(A_{\text{control}}\_A_{\text{sample}}\right)\right]/A_{\text{control}}]\times 100$$

where the *A*_control_ and *A*_sample_ determined the amount of control absorption and sample absorption, respectively (Sarafraz Ardakani et al., 2023).

#### 2.4.6 FRAP assay

The FRAP reagent was prepared by mixing acetate buffer (PH: 3.6), ferric chloride (20 mM), and 2,4,6‐triyridyl‐s‐triazine (TPTZ 10 mM in 40 mM HCL) in a ratio of 10:1:1. The FRAP reagent (3 mL) was added to extracted samples (150 μL) and placed at room temperature for 6 min. Then, the absorbance of samples was measured at 593 nm against methanol as a blank. The results were expressed by a standard curve of different concentrations of FeSO_4_.7H_2_O (mM Fe_2_SO_4_/100 g) (Sarafraz Ardakani et al., 2023).

#### 2.4.7 Saponin content

Approximately, 1 g of each sample was solved in 80% ethanol (20 mL) and heated in a water bath at 60°C for 1 h. Then, samples were centrifuged at 6251 *g* for 5 min, and the supernatant was passed through filter paper and concentrated at room temperature for 16 h. About 750 μL of extracted samples was mixed with 3 mL of glycolic acid/sulfuric acid in a ratio of 1:1. The absorbance of samples was recorded at 527 nm after 30 min. The total saponin content (SC) was expressed as g/100 g of oleanolic acid equivalent per 100 g dry sample (Lim et al., 2020).

#### 2.4.8 Phytic acid content

To measure phytic acid, the grounded samples (0.5 ± 0.05 g) were added to 10 mL of 2.4% (0.64 N) hydrochloric acid and vortexed for 10 s. The solution was left in the shaker for 16 h (overnight) at 300 rpm at room temperature. Then, the samples were centrifuged at 1512 *g* at 10°C for 20 min. The supernatant was filtered through Whatman filter paper into the tubes with the previously weighed NaCl (1.0 ± 0.05 g), and shaken for 20 min at 1512 *g*. The samples were allowed to settle at −20°C for 20 min. Then, the samples were centrifuged at 1512 *g* for 20 min. A quantity of 1 mL of the supernatant was diluted to 25 mL with deionized distilled water; the diluted sample (3 mL) was mixed with 1 mL of the Wade reagent (0.03% FeCl_3_∙6H_2_O + 0.3% sulfosalicylic acid), and centrifuged at 1512 *g* for 10 min. Finally, the absorbance of the reaction was recorded at 500 nm. The phytic acid content of the samples was expressed as milligrams of phytic acid per 100 g of dry sample (Darambazar, 2018).

#### 2.4.9 Statistical analysis

The data were analyzed by one‐way analysis of variance (ANOVA) using SPSS statistical software (SPSS 21.0 for Windows, SPSS Inc.). Statistical significance was considered at *p ≤* .05 and results were expressed as mean ± SD.

## 3 RESULTS AND DISCUSSION

The efficiency of common fermentation with *Saccharomyces cerevisiae* and fermentation using *L. casei* and *L. plantarum* on nutritional properties of quinoa was investigated by pH, acidity, total phenolic content, total flavonoids content, antioxidant capacity (FRAP, DPPH), saponin, and phytic acid parameters.

### 3.1 Titratable acidity

Titratable acidity (TA) determines the concentration of total acid in food through its complete titration with a standard base and is considered as a better predictor of the effect of organic acid on taste (Sadler & Murphy, 2010). The TA of fermented samples using different fermentation processes is given in Table 1. Results indicated that TA ranges are 5.15%–9.96%, 5.26%–13.53%, 4.94%–12.17%, and 4.38%–12.37% for F1, F2, F3, and F4, respectively. Despite no significant difference (*p >* .05) in TA between all fermented samples during the first 8 h (1–8 h), strains have been shown to behave differently through the incubation process. In other words, incubation time seems to be significant on TA value by different strains used for fermentation. In this regard, the F1 (i.e., samples fermented by *Saccharomyces cerevisiae*) showed the lowest level of acidity. In other words, the sample fermented by *Saccharomyces cerevisiae* had a lower acid production rate as reported by Bottani et al. (2018). Among samples fermented by LAB, F2 (i.e., samples fermented by *L. casei*) had the highest efficiency in acid production during 24‐h incubation. No significant difference (*p* > .05) has been observed between F3 and F4 in terms of TA. In the F4 sample which is the combination of *L. casei* and *L. plantarum* strains, it seems that the *L. plantarum* strain had a more decisive role in the fermentation process. The dominance of *L. plantarum* in a competitive environment has been previously stated by Teleky et al. (2020). Despite the changes observed in TA value during the fermentation time, no significant difference has been found in pH (data are not shown). This behavior has been attributed to the higher production of lactic acid compared to acetic acid (Nasiri Esfahani et al., 2018). The pH value was also decreased gradually during fermentation (data not shown), which is in accordance with Delgado et al. (2019), Carciochi et al. (2016), and Rizzello et al. (2016).

**TABLE 1:** Titratable acidity (%) of fermented samples during 24‐h fermentation.

Samples	Time (h)						
	1	4	8	12	16	20	24
F1	5.15 ± 0.21^a,D^	5.39 ± 0.13^a,D^	5.82 ± 0.08^c,C^	6.62 ± 0.32^b,B^	6.68 ± 0.05^c,B^	6.69 ± 0.07^c,B^	7.16 ± 0.12^c,A^
F2	5.26 ± 0.12^a,F^	5.74 ± 0.28^a,E^	6.68 ± 0.15^b,D^	6.99 ± 0.22^ab,D^	8.16 ± 0.14^b,C^	11.83 ± 0.12^a,B^	13.53 ± 0.11^a,A^
F3	4.94 ± 0.35^a,F^	5.82 ± 0.25^a,E^	7.46 ± 0.33^a,D^	7.50 ± 0.12^a,D^	9.93 ± 0.38^a,C^	11.55 ± 0.18^a,B^	12.17 ± 0.13^b,A^
F4	4.38 ± 0.04^b,G^	5.43 ± 0.22^a,F^	6.81 ± 0.18^b,E^	7.30 ± 0.18^a,D^	8.22 ± 0.03^b,C^	9.16 ± 0.00^b,B^	12.37 ± 0.07^b,A^

*Note*: The values presented are expressed as mean ± standard deviation of triplicate experiments. Different lowercase letters mean significant difference based on the Bonferroni test in each column; *p ≤* .05. Different capital letters mean significant difference based on the Bonferroni test in each row; *p ≤* .05. F1, The fermented samples by *Saccharomyces cerevisiae*; F2, The fermented samples by *L. casei*; F3, The fermented samples by *L. plantarum*; F4, The fermented samples by inoculation of *L. casei* and *L. plantarum* strains.

According to the results, the reduction in pH and acid production by LAB was more intensive than *Saccharomyces cerevisiae*. It is probably attributed to the growth and metabolic capacity of LAB and their ability to produce different levels of organic acids, especially lactic acid and acetic acid, during the fermentation process (Bottani et al., 2018; Lim et al., 2018).

### 3.2 Total phenolic and flavonoid contents

Phenolic compounds and flavonoids are secondary metabolites that are present in plants and considered two key indicators of the overall antioxidant capacity of the sample (Muhtadi & Wiyono, 2021). It was indicated that many microorganisms have the potential to release and metabolize secondary metabolites during fermentation (Melini & Melini, 2021). Phenolic compounds are found to be influenced by different factors such as pH and acidity, temperature, fermentation time, and the type of strain used in fermentation (Hashemi et al., 2019; Juan & Chou, 2010).

The results of the total phenolic content (TPC) and total flavonoid content (TFC) of the fermented samples are shown in Table 2. As can be seen, the TPC is reported for F1, F2, F3, and F4 in the range of 12.18–26.58, 12.18–28.34, 12.18–32.95, and 12.18–30.40 mg GAE/100 g, respectively. Also, TFC was in the range of 14.25–25.11, 14.25–24.93, 14.25–22.38, and 14.25–23.56 mg rutin/100 g for F1, F2, F3, and F4, respectively. Despite no significant difference (*p >* .05) in TPC and TFC of differently fermented samples at the beginning of fermentation (time 0), the incubation time was found to change them significantly (*p ≤* .05). Although an increase in parameters has been found during the first fermentation time of 12 h, a slight decrease was reported after that. In other words, the maximum quantity of TPC and TFC of fermented samples was observed during the early fermentation stages (8–12 h), while the maximum amount of TPC and TFC in F1 and F2 was observed after 8 h and in F3 and F4 after 12‐h incubation. Generally, among the differently fermented samples, F3 (i.e., samples fermented by *L. plantarum*) shows the highest increase in TPC content (170.52%) after 12‐h fermentation. In other words, the ability of *L. plantarum* to increase and release TPC was significantly (*p ≤* .05) higher than F1, F2, and F4, with values equal to 118.22%, 132.67%, and 149.58%, respectively. The highest concentration of TFC was also observed in the F1 and F2 (76.21% and 74.94%, respectively) after 8‐h fermentation. This indicates the efficiency of F1 and F2 to increase the flavonoid compounds significantly (*p ≤* .05) higher than F3 and F4 (57.05% and 65.33%, respectively). The initial increase in TPC and TFC during 8–12 h of incubation is probably attributed to changes induced by the fermentation process including the release of hydrolytic enzymes and also a decrease in pH, which may provide optimal pH conditions for native hydrolytic enzymes such as β‐glucosidase, α‐amylase, cellulase, inulinase, and esterase. These enzymes are potentially able to break down the cell wall structure and hydrolyze the ester bonds. As a result of the activity of these enzymes (especially the β‐glucosidase), phenolic compounds bound with glycosides are converted into their free forms, which are more bioavailable (Juan & Chou, 2010; Melini & Melini, 2021; Nisa et al., 2019). These results are in accordance with Adebo et al. (2018) and Ryu et al. (2019) investigations.

**TABLE 2:** Total phenolic content and total flavonoid content of fermented samples during 24‐h fermentation.

Parameters	Samples	Time (h)						
		0	4	8	12	16	20	24
TPC	F1	12.18 ± 0.18^a,F^	19.18 ± 0.04^c,D^	26.58 ± 0.45^b,A^	25.80 ± 0.30^c,AB^	25.42 ± 0.23^c,B^	23.14 ± 0.20^b,C^	18.13 ± 0.23^c,E^
	F2	12.18 ± 0.18^a,F^	20.58 ± 0.19^b,E^	28.34 ± 0.46^a,A^	26.66 ± 0.50^c,B^	25.09 ± 0.30^c,C^	22.71 ± 0.32^b,D^	20.99 ± 0.30^b,E^
	F3	12.18 ± 0.18^a,G^	18.60 ± 0.30^c,F^	23.92 ± 0.58^d,E^	32.95 ± 0.42^a,A^	29.73 ± 0.45^a,B^	25.76 ± 0.16^a,C^	22.45 ± 0.28^a,D^
	F4	12.18 ± 0.18^a,F^	23.03 ± 0.22^a,D^	25.71 ± 0.15^c,C^	30.40 ± 0.34^b,A^	28.19 ± 0.27^b,B^	20.96 ± 0.32^c,E^	20.61 ± 0.39^b,E^
TFC	F1	14.25 ± 0.24^a,E^	20.39 ± 0.43^a,C^	25.11 ± 0.57^a,A^	25.09 ± 0.02^a,A^	22.42 ± 0.23^a,B^	19.63 ± 0.06^a,D^	20.87 ± 0.29^a,C^
	F2	14.25 ± 0.24^a,D^	19.00 ± 0.76^b,C^	24.93 ± 0.71^a,A^	19.60 ± 0.45^d,C^	20.95 ± 0.34^b,B^	20.06 ± 0.64^a,BC^	18.72 ± 0.76^b,C^
	F3	14.25 ± 0.24^a,E^	19.41 ± 0.74^b,C^	19.87 ± 0.58^c,C^	22.38 ± 0.57^c,A^	20.95 ± 0.34^b,B^	19.13 ± 0.36^a,CD^	18.07 ± 0.28^b,D^
	F4	14.25 ± 0.24^a,F^	17.01 ± 0.23^c,E^	20.71 ± 0.15^b,B^	23.56 ± 0.12^b,A^	19.54 ± 0.06^c,C^	16.17 ± 0.78^b,E^	18.49 ± 0.32^b,D^

*Note*: The values presented are expressed as mean ± standard deviation of triplicate experiments. Different lowercase letters mean significant difference based on the Bonferroni test in each column; *p ≤* .05. Different capital letters mean significant difference based on the Bonferroni test in each row; *p ≤* .05. F1, The fermented samples by *Saccharomyces cerevisiae*; F2, The fermented samples by *L. casei*; F3, The fermented samples by *L. plantarum*; F4, The fermented samples by inoculation of *L. casei* and *L. plantarum* strains.

A slight decrease in TPC and TFC after 12‐h fermentation may be attributed to the abstraction of hydride ions and rearrangement of phenolic structure induced by the reduction of pH (Adebo et al., 2018). Besides, elucidates existed that decrease in phenolic compounds during the lactic fermentation of cereals could be attributed to the release of phenolic compounds into cellular fluids and the activation of polyphenol oxidase enzyme during the soaking process (Olaniyi & Mehdizadeh, 2013). This enzyme is claimed to accelerate the oxidation of polyphenols, leading to decreased total phenolic content (Cuellar‐Álvarez et al., 2017). Increased bonding and interaction of phenolic compounds and flavonoids with macromolecules such as proteins were also reported as another probable reason for TPC reduction (Adebo & Gabriela Medina‐Meza, 2020; Tian, Liu, et al., 2018).

### 3.3 Antioxidant capacity

In the present study, two different methods (DPPH and FRAP) were used to determine the antioxidant capacity and are represented in Table 3. The principle of DPPH method is based on the reduction of DPPH radical to diphenylpicrylhydrazyl by antioxidants. DPPH is able to determine the hydrophilic and lipophilic antioxidant compounds, while the FRAP method is based on the reduction of the ferric tripyridyltriazine (Fe (III)‐TPTZ) complex to ferric tripyridyltriazine (Fe (II)‐TPTZ) by antioxidants. Despite the DPPH method, FRAP is only able to determine the hydrophilic antioxidant compounds (Muhtadi & Wiyono, 2021; Szydłowska‐Czerniak & Łaszewska, 2015). The increase in antioxidant capacity through the fermentation process is influenced by various factors such as the type of microorganism, pH, temperature, fermentation time, and the type of substrate (Hur et al., 2014). Considering the results, the range of antioxidant capacity by FRAP method was 19.02–31.19, 19.02–31.11, 19.02–38.93, and 19.02–35.31 mM Fe_2_SO_4_/100 g for F1, F2, F3, and F4, respectively. Also, antioxidant capacity by the DPPH method was in the range of 71.77–91.52, 71.77–91.67, 71.77–94.18, and 71.77–93.49 g/100 g for F1, F2, F3, and F4, respectively.

**TABLE 3:** Antioxidant capacity (FRAP and DPPH) of fermented samples during 24 h of fermentation.

Antioxidant assay	Samples	Time (h)						
		0	4	8	12	16	20	24
FRAP	F1	19.02± 0.48^a,E^	24.55 ± 0.32^b,D^	31.19 ± 0.33^b,A^	30.80 ± 0.19^c,A^	30.07 ± 0.33^c,B^	29.93 ± 0.17^c,B^	25.81 ± 0.61^c,C^
	F2	19.02 ± 0.48^a,D^	22.05 ± 0.41^d,C^	31.11 ± 0.41^b,A^	30.97 ± 0.08^c,A^	31.26 ± 0.16^b,A^	30.84 ± 0.25^b,A^	27.71 ± 0.80^b,B^
	F3	19.02 ± 0.48^a,F^	23.02 ± 0.10^c,E^	23.05 ± 0.79^c,E^	38.93 ± 0.76^a,A^	35.44 ± 0.44^a,B^	33.36 ± 0.19^a,C^	30.97 ± 0.82^a,D^
	F4	19.02 ± 0.48^a,G^	25.44 ± 0.49^a,D^	32.78 ± 0.08^a,B^	35.31 ± 0.07^b,A^	28.78 ± 0.64^d,C^	23.07 ± 0.04^d,F^	24.10 ± 0.12^d,E^
DPPH	F1	71.77 ± 0.07^a,G^	89.03 ± 0.37^b,D^	91.52 ± 0.19^b,A^	91.10 ± 0.03^c,B^	91.73 ± 0.08^c,C^	88.01 ± 0.22^a,E^	86.22 ± 0.05^a,F^
	F2	71.77 ± 0.07^a,F^	89.23 ± 0.14^b,D^	91.67 ± 0.16^ab,A^	91.12 ± 0.05^c,C^	91.30 ± 0.36^a,B^	89.15 ± 0.10^b,D^	86.21 ± 0.35^a,E^
	F3	71.77 ± 0.07^a,E^	85.89 ± 0.28^c,C^	88.41 ± 0.30^c,B^	94.18 ± 0.31^a,A^	88.27 ± 0.42^d,B^	86.00 ± 0.13^c,C^	82.32 ± 0.48^b,D^
	F4	71.77 ± 0.07^a,E^	91.67 ± 0.10^a,B^	91.81 ± 0.08^a,B^	93.49 ± 0.01^b,A^	90.94 ± 0.09^b,C^	86.98 ± 0.06^d,D^	86.82 ± 0.22^a,D^

*Note*: The values presented are expressed as mean ± standard deviation of triplicate experiments. Different lowercase letters mean significant difference based on the Bonferroni test in each column; *p ≤* .05. Different capital letters mean significant difference based on the Bonferroni test in each row; *p ≤* .05. F1, The fermented samples by *Saccharomyces cerevisiae*; F2, The fermented samples by *L. casei*; F3, The fermented samples by *L. plantarum*; F4, The fermented samples by inoculation of *L. casei* and *L. plantarum* strains.

According to the results, incubation time has been found to significantly (*p ≤* .05) influence the antioxidant capacity on the basis of the strain used in its fermentation. Overall, an initial increase in antioxidant capacity has been found during the first 8–12 h of incubation which has decreased slightly. In this regard, maximum antioxidant capacity (FRAP, DPPH) was reported during 8–12 h of fermentation, with the maximum antioxidant capacity of F1 and F2 after 8 h and F3 and F4 after 12 h of fermentation. The highest increase in antioxidant capacity by the FRAP method is observed at F3 (i.e., samples fermented by *L. plantarum*) with values of 63.98%, 63.56%, 104.67%, and 85.64 for F1, F2, F3, and F4, respectively. Also, the highest radical scavenging activity (DPPH) was related to the F3 (i.e., samples fermented by *L. plantarum*), in which rate of increase in F1, F3, F3, and F4 was 27.51%, 27.21%, 31.22%, and 30.26%, respectively.

Antioxidant capacity indicates the presence of various antioxidant compounds, including phenolic compounds (the highest capacity), carotenoids, betacyanins, and tocopherols (Vosoughi et al., 2018). The fermentation process is reported to positively influence the antioxidant capacity either by the increase in phenolic and/ or flavonoid compounds (induced by microbial hydrolysis reaction) or their synthesis by microorganisms (Carciochi et al., 2016; Hur et al., 2014). In addition, a slight decrease in antioxidant capacity after 12 h may be related to oxidation and a reduction of antioxidant compounds such as TPC (Zhou et al., 2020), which is concurrent with Robbins (2003) and de Souza et al. (2018) findings. The results of the current research show that the antioxidant capacity (FRAP and DPPH), TPC, and TFC have similar trends during fermentation, which indicates that the presence of phenolic compounds potentially had a direct role in the antioxidant capacity (Dudonne et al., 2009). Also, Lim et al. (2020) and Ibrahimi and Hajdari (2020) reported a positive relationship between phenolic and flavonoid compounds with both antioxidant assay systems (FRAP and DPPH).

### 3.4 Saponin content

Saponins are bitter compounds that are naturally present in quinoa seeds in the range of 0.1 to 5 g/100 g. The presence of saponins in quinoa seeds has adverse effects on the bioavailability of vitamins and minerals (Karovičová et al., 2020). Therefore, the removal of saponins from quinoa seeds is considered a necessary step before consumption. Saponins are phytochemical compounds that have at least one glycosidic bond between their aglycone and sugar chains (El Aziz et al., 2019). It is reported that during the fermentation by microorganisms such as lactic acid bacteria and *Saccharomyces cerevisiae* under optimal pH conditions may produce enzymes such as β‐glucosidases (Bonciani et al., 2018; Nisa et al., 2019). The β‐glucosidases can break down the sugar side chains of steroid and triterpenoid saponins and reduce the water solubility of these compounds (Rui et al., 2017). As a result, this reaction probably leads to the breakdown of the saponin structure during fermentation. Moreover, the reduction of saponin during fermentation is affected by various factors such as fermentation time, the type of microorganism used in fermentation, and the pH of the samples (Sharath et al., 2014; Tian, Na, et al., 2018).

**FIGURE 1:**
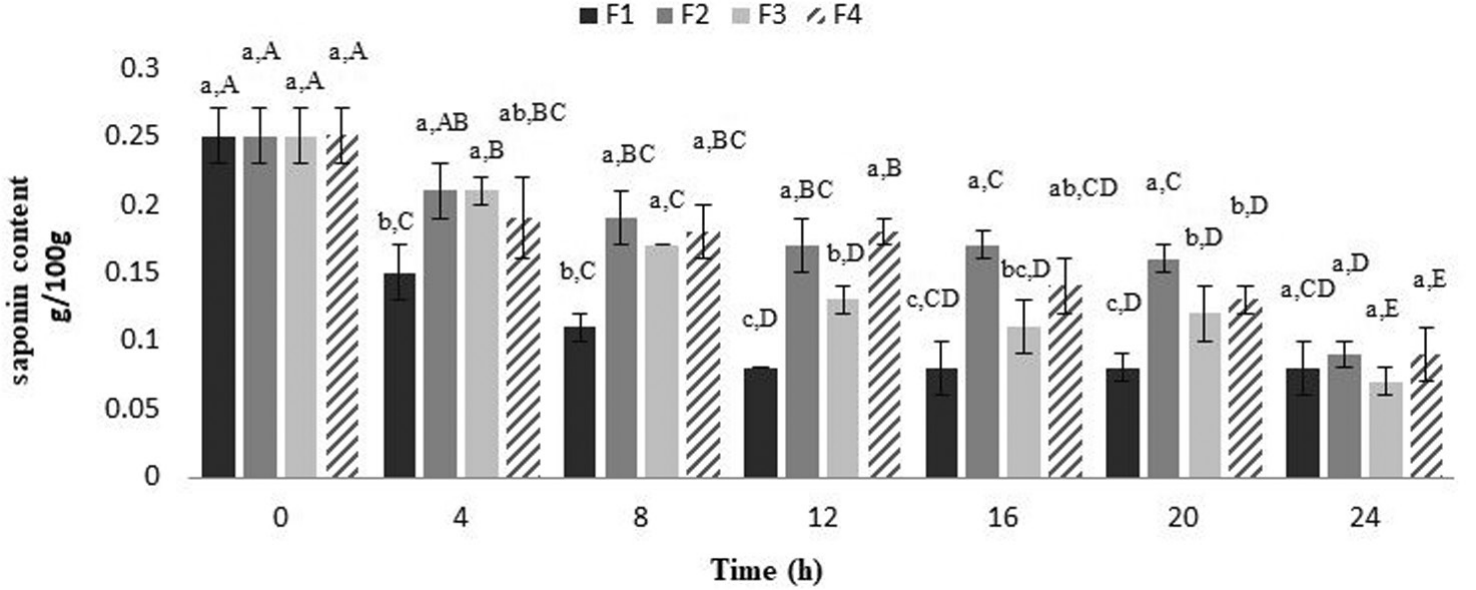
Saponin content of fermented samples during 24‐h fermentation. *The values presented are expressed as mean ± standard deviation of triplicate experiments. **Different lowercase letters mean significant difference based on the Bonferroni test in each column; *p ≤* .05. ***Different capital letters mean significant difference based on the Bonferroni test in each row; *p ≤* .05. ****F1, The fermented samples by *Saccharomyces cerevisiae*; F2, The fermented samples by *L. casei*; F3, The fermented samples by *L. plantarum*; F4, The fermented samples by inoculation of *L. casei* and *L. plantarum* strains.

As shown in Figure 1, the saponin content range of the fermented samples was between 0.25–0.08, 0.25–0.09, 0.25–0.07, and 0.25–0.09 g/100 g for F1, F2, F3, and F4, respectively. Results indicated that increasing the incubation time can significantly reduce the amount of SC in strain‐dependent manner. In other words, *Saccharomyces cerevisiae* was found as the most efficient strain in saponin reduction in short‐time fermentation processes in which 68.99% reduction has been found only after 12‐h incubation. However, similar reduction efficiency is found at the end of the fermentation process with a reduction ratio of 68.99%, 64.11%, 72.86%, and 64.11% by F1, F2, F3, and F4, respectively. It was indicated that the reduction in SC during fermentation is due to the LAB glycosylation process (Michlmayr & Kneifel, 2014). During this process, glycosyltransferases provide water solubility and chemical stability for aglycones. Diglycosides attached to aglycone are hydrolyzed by specific glycosidases. Then, aglycones are released due to the activity of mono glucosidases such as β‐glucosidases. Finally, the saponin structure breaks down and loses its bitterness (J. G. Lim et al., 2020). Also, the highest efficiency of *Saccharomyces cerevisiae* in saponin reduction may be attributed to the difference in the optimal pH for the β‐glucosidase activity (Yuksekdag et al., 2017), which is reported at 5.5 and 4–5 for *Saccharomyces cerevisiae* and LAB, respectively (Carciochi et al., 2016; Sestelo et al., 2004; Yuksekdag et al., 2017).

### 3.5 Phytic acid contents

Phytate is the main storage form of phosphorus in mature grains, but it is considered an antinutritional agent, because it can form complexes with minerals, starch, and proteins, thereby limiting their bioavailability (Feizollahi et al., 2021). The reduction of phytic acid content during fermentation depends on various factors that can contribute to phytate degradation including pH, phytase activity, temperature, fermentation time, as well as the starter type (Anastasio et al., 2010). The phytic acid content of fermented samples using different fermentation processes is given in Table 4. The range of phytic acid content in the fermented samples in the present study was between 203.17–106.49, 203.17–106.40, 203.17–71.84, and 203.17–88.72 mg/100 g for F1, F2, F3, and F4, respectively. Results indicated that all fermented samples significantly (*p ≤* .05) reduced the phytic acid content during 24‐h fermentation, and a scattered increase in phytic acid in all samples was observed. Generally, the maximum reduction of phytic acid in F1 samples was reported after 8‐h incubation (47.58%), while it was continued up 16 h for F4 (56.33%) and to 24 h for F2 and F3 samples with values of 47.63% and 64.64% reduction, respectively. In addition, it was found that during the first 8 h of fermentation (0–8 h), samples F1 (i.e., samples fermented by *saccharomyces cerevisiae*) were more efficient in reducing phytic acid compared to other ones. However, with an increase in the fermentation process up to 24 h, F3 (samples fermented by *L. plantarum*) shows the highest reduction in phytic acid compared to other samples.

**TABLE 4:** Phytic acid content of fermented samples during 24‐h fermentation.

Samples	Time						
	0	4	8	12	16	20	24
F1	203.17 ± 0.23^a,A^	159.23 ± 0.70^b,D^	106.49 ± 0.19^c,G^	196.47 ± 0.89^a,B^	129.35 ± 0.73^b,E^	125.60 ± 0.57^c,F^	164.19 ± 0.65^a,C^
F2	203.17 ± 0.23^b,A^	138.53 ± 0.45^d,D^	120.46 ± 0.26^b,E^	180.56 ± 0.94^b,B^	151.45 ± 0.57^a,C^	116.42 ± 0.78^d,F^	106.40 ± 0.92^c,G^
F3	203.17 ± 0.23^c,A^	150.16 ± 0.29^c,B^	121.99 ± 0.94^b,D^	88.38 ± 0.52^d,E^	79.07 ± 0.53^d,F^	143.09 ± 0.73^b,C^	71.84 ± 0.90^d,G^
F4	203.17 ± 0.23^d,A^	177.41 ± 0.63^a,B^	140.83 ± 0.62^a,E^	146.34 ± 0.51^c,D^	88.72 ± 0.40^c,G^	150.81 ± 0.95^a,C^	129.31 ± 0.64^b,F^

*Note*: The values presented are expressed as mean ± standard deviation of triplicate experiments. Different lowercase letters mean significant difference based on the Bonferroni test in each column; *p ≤* .05. Different capital letters mean significant difference based on the Bonferroni test in each row; *p ≤* .05. F1, The fermented sample by *Saccharomyces cerevisiae*; F2, The fermented sample by *L. casei*; F3, The fermented sample by *L. plantarum*; F4, The fermented sample by inoculation of *L. casei* and *L. plantarum* strains.

Studies have shown that the reduction of phytate during fermentation is probably related to the enzymatic activity of microorganisms and endogenous phytase (Olukomaiya et al., 2020). In the optimum pH (4.5–5.5), fermentation products and fermentation temperature provide favorable conditions for the activity of the phytases (Castro‐Alba, Lazarte, Perez‐Rea, Sandberg, et al., 2019). Castro‐Alba, Lazarte, Perez‐Rea, Sandberg, et al. (2019) stated that phytate reduction in quinoa flour is firstly done by its endogenous phytases and followed by exogenous phytase enzymes produced by microorganisms (Castro‐Alba, Lazarte, Perez‐Rea, Carlsson, et al., 2019). The better efficiency of *Saccharomyces cerevisiae* in phytic acid reduction is probably induced by its faster speed in providing favorable conditions (Leenhardt et al., 2005). Among the LAB strains, the highest removal of phytic acid content was observed in the sample containing *L. plantarum*, which is also consistent with the findings of Hashemi et al. (2019) which is probably attributed to the higher phytase activity of *L. plantarum* compared to *L. casei*.

Sporadically increasing trend of phytic acid after 8‐h incubation may be related to the release and leakage of more phytic acid enclosed in bran and endosperm of quinoa during fermentation (Mehanni et al., 2017; Servi et al., 2008). Soaking is a standard step in the fermentation process that can reduce the levels of various antinutrients because many antinutrient compounds, such as phytate, are water‐soluble and can be removed better by leaching (Samtiya et al., 2020). During soaking, phytate ions leak into the water in the direction of the concentration gradient, and with the longer soaking period, more phytate ions leak into the environment (Mehanni et al., 2017; Servi et al., 2008). On the other hand, it is possible that protein‐bound phytates are released due to the activity of protease enzymes (Chen et al., 2014). It has also been reported that by increasing the soaking time, the action of phytase enzymes decreased by about 10%–60% (Feizollahi et al., 2021). Therefore, an increase in the amount of phytic acid is observed. However, at the end of fermentation (24 h) time, phytic acid was still observed in the range of 164.19–71.84 mg/100 g in all samples. As the fermentation is carried out for a more extended period, the reduction of phytic acid would be observed. In other studies, it has been mentioned that the complete removal of phytic acid is possible within 48 h (Chen et al., 2014).

## 4 CONCLUSIONS

In the present study, the potential of two fermentation processes to reduce antinutritional compounds and improve the nutritional value (antioxidant capacity) of quinoa flour has been investigated. The results showed that the incubation time based on the type of strain used in fermentation significantly (*p ≤* .05) influenced antinutritional factors (phytic acid and saponin), as well as the antioxidant capacity of quinoa dough by changing the content of total phenolic and flavonoids during the fermentation process. In fact, changes in pH and production of acidity during fermentation create favorable conditions for the activities of endogenous enzymes of quinoa and enzymes produced by microorganisms. In the current study, all samples have been able to reduce saponin by 66% on average after 24‐h fermentation. Among strains used for fermentation, *L. plantarum* showed the maximum reduction of phytic acid content after 24 h. It should be noted that *Saccharomyces cerevisiae* has a higher efficiency in reducing antinutritional factors (saponin, phytic acid) in short periods (0–8 h) than other strains. Also, the maximum increase in TPC and antioxidant capacity (FRAP and DPPH) in samples fermented by *L. plantarum* after 12 h of fermentation was reported. Also, the highest concentration of TFC was observed in the samples fermented by *Saccharomyces cerevisiae* and *L. casei* after 8‐h fermentation. Therefore, *Lactiplantibacillus plantarum* could be used to reduce the antinutritional factors as well as improve the antioxidant properties of quinoa dough during fermentation. Also, fermentation by the combination of *Saccharomyces cerevisiae* and *L. plantarum* is suggested for future research.

## AUTHOR CONTRIBUTIONS

**Sanaz Arjmand:** Conceptualization (equal); data curation (equal); investigation (equal); project administration (equal); resources (equal); writing – original draft (equal); writing – review and editing (equal). **Neda Mollakhalili‐Meybodi:** Conceptualization (equal); data curation (equal); funding acquisition (equal); methodology (equal); resources (equal); supervision (equal); visualization (equal); writing – review and editing (equal). **Fateme Akrami Mohajeri:** Conceptualization (equal); formal analysis (equal); funding acquisition (equal); methodology (equal); project administration (equal); resources (equal); supervision (equal); visualization (equal); writing – review and editing (equal). **Farzan Madadizadeh:** Data curation (equal); formal analysis (equal); methodology (equal); resources (equal); software (equal); validation (equal); visualization (equal); writing – review and editing (equal). **Elham Khalili Sadrabad:** Conceptualization (equal); funding acquisition (equal); investigation (equal); project administration (equal); resources (equal); supervision (equal); validation (equal); writing – review and editing (equal).

## FUNDING INFORMATION

This research project has been financially supported by Shahid Sadoughi University of Medical Sciences, Yazd.

## CONFLICT OF INTEREST STATEMENT

All authors declare that they have no conflict of interest.

## ETHICS STATEMENT

The current research does not contain human or animal studies.

## Data Availability

Data are available on request from the authors.
